# A civil society view of rare disease public policy in six Latin American countries

**DOI:** 10.1186/s13023-020-1314-z

**Published:** 2020-02-27

**Authors:** Mo Mayrides, Eva Maria Ruiz de Castilla, Silvina Szelepski

**Affiliations:** 1 Emoluva Partners, LLC, 10142 SW 79th Avenue, Miami, FL 33156 USA; 2 Latin America Patients Academy, Miami, FL USA; 3 Knight & Pawn, Miami, FL USA

**Keywords:** Rare diseases, Orphan drugs, National laws, Policy, Legislation, Regulations, Patient advocacy, Patient engagement, Health system incorporation, Latin America

## Abstract

Patients with rare diseases across the world struggle to access timely diagnosis and state-of-the-art treatment and management of their conditions. Several recently published reviews highlight the importance of country efforts to address rare diseases and orphan drugs policy comprehensively. However, many of these reviews lack depth and detail at the local level, which we believe is necessary for rare disease advocates to identify and prioritize opportunities for strengthening each country’s policy framework.

We asked leading patient advocates from civil society organizations their views on rare disease public policy in Argentina, Brazil, Chile, Colombia, Mexico, and Peru with a focus on whether specific laws and regulations in these six Latin American countries have been promulgated. From December 2018 to March 2019 we supplemented their perspectives with evidence from accessible literature using key search terms. For each country, we prepared a detailed analysis on how laws or other policy initiatives took shape and the steps taken since to implement them. This allowed us to identify five broad policy categories for subsequent analysis: national laws, national regulations, health system incorporation of rare disease treatments, care delivery, and patient engagement.

By describing the different approaches, challenges and timelines across six countries, our research demonstrates that strengthening rare disease policy first requires a common understanding and local consensus of each country’s recent past and current situation. Subsequent analysis based on a set of common policy dimensions led us to where we believe salient opportunities lie for each of these countries to strengthen their overall policy framework for rare disease patients.

## Background

Rare diseases are known to often impact patients from the time of birth, affect multiple organ systems, are severely disabling, reduce life expectancy, and impair physical and mental abilities. Due to their low prevalence, unique and coordinated efforts are necessary to address quality of life and prevent significant early mortality and morbidity. Distinct challenges for rare disease patients include early and accurate diagnosis, as well as access to effective treatment. Policy makers, patients and payers struggle with costly treatments and inadequate care coordination and infrastructure. Most medical professionals have very limited knowledge of rare diseases unless they are specialized in certain disease areas [1].

As a result, many more countries worldwide are recognizing the need to address rare diseases and orphan drug policy comprehensively. In a literature review of policies for orphan drug access in 35 countries, researchers found that 27 have orphan drug legislation in place, 18 have national plans, 26 offer incentives for orphan drug research and development, and 33 provide for official reimbursement of orphan drugs [2]. An 11-country analysis of rare disease policies found that most have plans or at least intend to develop national plans to address rare diseases [3]. Researchers found that even those countries without formal national plans had developed some policies to address health care access and/or services for patients with rare diseases.

This review explores rare disease public policy in Argentina, Brazil, Chile, Colombia, Mexico, and Peru. The challenges across Latin America are similar to other parts of the world in that patients still struggle to access timely diagnosis and state-of-the-art treatment and management of their conditions. Meanwhile, the regulatory frameworks and legal protections in Latin America are relatively new as decision makers generally lack reliable information and have only recently become more aware of the unique challenges posed by rare diseases [4]. The six countries we studied were also included in a recent rare disease policy global review of the literature with results published in November 2018 [5].

## Methodology

We wanted to take this kind of research to a new level of detail. Specifically, we wanted to know whether specific laws and regulations in these six countries in Latin America had been promulgated and if so, the current environment and focus for policy implementation in each country. Updated and specific information on rare disease policy implementation in each country will help our respective stakeholder communities identify important gaps and address unmet needs.

We gathered information from leading patient advocates for rare disease patients in each of the countries studied via telephone and during the course of face-to-face patient advocacy meetings in December 2016, March 2017, July 2017, October 2017, December 2017, and December 2018. It was also important to match the experiences and perspectives that were shared with evidence from accessible literature using key search terms in PubMed Central, Google Scholar, general online searches using Google, reviews of international and local patient advocacy organization websites, public legislative databases, and article searches in academic specialty journals such as the *Pan-American Journal of Public Health*. Abstracts from literature search results were scanned to determine usefulness and relevance to the research. In a few cases where access to a full publication was limited, we directly contacted authors via email to obtain the studies of interest. This desktop research was conducted from December 2018 through March 2019 and included review of publications and other available resources in either English, Spanish, or Portuguese.

For each country, we prepared a detailed analysis beginning with national laws or rare disease regulations in countries where official laws are not yet on the books. How these laws or initiatives took shape and the steps taken since to implement them allowed us to identify five broad policy categories for subsequent analysis. These five categories are detailed in Table 1. Analysis among the six countries using these categories led us to where we believe the greatest opportunities lie for each of these countries to strengthen their overall policy framework for rare disease patients.

**Table 1:** Policy Categories for Analysis

Policy Category	Description
National Laws	Existence of a national law specific to rare diseases
National Regulations	Regulations published (or drafted) by the government to implement the national law
Health System Incorporation	Inclusion of rare diseases and treatments in health system financing and reimbursement programs
Care Delivery	Extent to which care models are utilized or proposed, such as rare disease centers of excellence or reference centers
Patient Engagement	Level of engagement by governments of patient advocates and their organizations

### National Laws

Colombia [6], Peru [7], Mexico [8], and Argentina [9] have adopted national laws passed by each’s Congress to specifically address rare diseases. These are described in Table 2. In Brazil, the government issued an ordinance in 2014 without national legislation [10], though there are as many as 14 bills pending in Congress to address different elements of rare disease policy [12]. In Chile, the 2015 Ricarte Soto [11] Law had originated with an exclusive focus on rare diseases, but was modified to provide financial protection for the broader category of high-cost diseases [13].

**Table 2:** National Laws on Rare Diseases in 6 Latin American Countries

Country	Law/Ordinance	Date Enacted	Description
Argentina	Law 26,689	June 29, 2011	Defines rare diseases in Argentina as those that affect 1 of every 2000 persons and dictates comprehensive care and coverage for rare disease patients. It mandates national patient- and disease-specific data registries, centers of excellence, interdisciplinary care for rare disease patients, expanded newborn screening programs, and research and development of health technologies to address rare diseases. Of note, in Argentina rare diseases are referred to in Spanish as “diseases of low prevalence” given the negative connotation of the word “rare.”
Brazil	Ordinance 199	January 30, 2014	Ordinance 199 is an administrative act (not passed by Congress). It calls for greater public awareness, improving diagnosis, increasing education and training among health professionals, expanding reference centers, and developing clinical protocols for prioritized rare diseases. Ordinance 199 expands a 2009 policy on clinical genetics [10].
Chile	Law 20.850	June 1, 2015	The Financial Protection System for High Cost Diagnostics and Treatments (Ricarte Soto Law) creates a program for diagnostic and treatment coverage for select high-cost diseases and, as such, facilitates access for some rare disease patients. The law originated with a focus on rare diseases, but is not specific to rare diseases [11].
Colombia	Law 1392	July 2, 2010	Mandates social protection for all with rare diseases and measures to address in a comprehensive manner the needs of these patients in all dimensions and in all relevant phases of disease awareness, diagnosis, prevention, treatment, and ongoing management. The law guarantees access to medicines and diagnosis, calls for health professional training in rare diseases, a data/information collection system, centers of excellence, and rare disease research networks [6].
Mexico	Article 224 bis and bis 1 of the General Health Law	January 30, 2012	Defines orphan medicines for rare diseases that affect < 5 individuals in 10,000 and mandates the nation’s Secretary of Health consider any and all means necessary to make these medicines available to the Mexican population [8].
Peru	Law 29698	June 4, 2011	Declares of national interest the prevention, diagnosis, and comprehensive treatment for persons with rare diseases. Calls on the Ministry of Health to adopt mechanisms to ensure orphan drugs are available to patients, develop a rare disease patient registry, build a national action plan, seek measures to improve diagnosis, and include rare diseases in medical education and training curricula. The law considers rare disease services and treatments in Peru a priority for annual health budgeting and resource allocation purposes [7].

Mexico’s law is the least descriptive; the nation revised its general health law in 2012 to define rare diseases based on incidence and to task the Health Secretary with ensuring the problem be addressed by any means. The legislatures in Peru and Argentina passed comprehensive national laws in 2011, but implementation is just beginning in both countries.

Only in Argentina is the national law being codified at the state or municipal level [14], although some cities in Colombia have used that country’s 2010 national law as a model for local policy [15, 16]. Colombia’s law is the oldest, while Chile’s Ricarte Soto Law the newest; the difference in approach is noteworthy. Colombia’s law is a social protection measure with rare disease patients considered a protected class. In Chile, only select rare disease patients are covered, and while financial protection is critical, additional policy elements for the rare disease community have yet to be considered.

There are opportunities in each of the six countries to improve the legal framework for rare disease patients. As discussed, in Chile the gaps in the Ricarte Soto Law present an opportunity for rare disease experts and advocates to argue for a legal framework specifically for rare diseases. In Argentina, Colombia, and Peru, these opportunities are mostly in governments following through with implementation regulations. In Mexico, there has been little in the way of concerted action to address rare diseases since the revision of the health law in 2012. Rare disease advocates in Brazil are advocating for Congress to wrap together several legislative proposals into one larger bill, which could be an important mechanism for modernizing that nation’s rare diseases administrative ordinance [12].

### National Regulations

Table 3 lists national regulations on rare diseases in the six countries. In Colombia, at least 6 implementing regulations [20–25] have been issued since the national law passed in 2010, although most are related to how the country identifies and officially registers rare diseases and patient caseload nationwide. Only recently is Colombia moving beyond this; in April 2018 the Health Ministry issued its first resolution on the arrangement of clinical care networks for rare disease patients [25]. Brazil’s ordinance is heavily focused on specialized care for rare disease patients within the Sistema Único da Saúde (SUS), but implementation has been slowed by the requirement that each rare disease have its own clinical protocol and therapeutic guideline (PCDT) [12]. More recent PCDTs and incorporations of rare disease medicines into SUS should provide new, positive momentum for the outlook in Brazil.

**Table 3:** National Regulations for Rare Diseases in 6 Latin American Countries

Country	Regulation(s)	Date Enacted	Description
Argentina	Resolution 2329	December 22, 2014	Creates national program for rare diseases and congenital anomalies. For rare diseases, specifies developing a list of these and a national patient registry. All other provisions such as advisory board, public awareness, training of public health professionals, and a national network of diagnosis and care centers apply to both rare diseases and congenital anomalies [17].
	Decree 794	May 11, 2015	Specifically regulates the national rare diseases law with provisions such as a national advisory board, studies to determine the existing care infrastructure, and minimum coverage standards. Most of the specific mandates in the national law are left “unregulated” in the decree [18].
–	Resolution 271/19	February 13, 2019	Establishes the Advisory Board for Rare Diseases and Congenital Anomalies to be led by the Ministry of Health and to include representatives from several hospitals, one medical society, and five patient-based organizations. Mandates the Board create rare diseases list for Ministry approval [19].
Brazil	N/A	–	See Ordinance 199
Chile	N/A	–	See Law 20.850
Colombia	Decree 1954	September 19, 2012	Describes the data collection mechanism on number of rare diseases in Colombia [20].
–	Resolution 430	February 20, 2013	Lists 1940 different rare diseases in the country [21]
–	Resolution 3681	September 19, 2013	Specifies how the government would collect information on rare disease patients via the high-cost account. Data from the one-time census of patients in 2013 was made public via the Ministry’s SISPRO system capturing data on just over 13,000 patients, including their age, gender, type of rare disease, and geographic residence [22]
–	Resolution 123	January 21, 2015	Mandates the continued reporting of hemophilia and related coagulopathies to the high-cost account. These were the only diseases mandated for continued reporting beyond the one-time census of rare disease patients in 2013 [23]
–	Resolution 2048	June 9, 2015	Updates the number of rare diseases in Colombia to 2149 with each assigned a number code (1–2149). The coding is important for use in health system settings with the goal of improving surveillance of rare diseases over time and nationwide [24]
–	Resolution 651	March 1, 2018	Outlines the processes, standards, and criteria for health centers to become officially recognized rare disease reference centers for diagnosis, treatment, and management. Also defines how these reference centers would then link to each other in networks and sub-networks to cover all of Colombia [25].
Mexico	N/A	–	–
Peru	Decree 004-2019-SA	February 22, 2019	Mandates national plan, rare disease patient registry, scientific and medical research, health personnel training, and coordinated care regardless of coverage status or source of medical care. Also mandates budget impact and other economic evaluation studies for high-cost rare disease diagnostic tests and medicines to be carried out by a new health technology evaluation agency called RENETSA. Main public payers are to establish consultative councils to review rare disease cases to recommend when treatment is warranted, but final decisions will be made by each payer depending on a budget impact analysis and funds availability [26].

Peru published a final regulation in February 2019 [26], but this version of rulemaking differs from earlier drafts and much remains to be defined as the Ministry of Health begins implementation. The rule lays out what seems to be a substantial bureaucracy for evaluating both new medicines and rare disease patient cases, so it may take significant time before impact can be evaluated. Based on the rule, however, it seems much in Peru will depend on budget impact studies and the availability of funds within the reimbursement structures of the main payer systems, which usually means new barriers to impede instead of expand patient access. Also, beyond reimbursement, there is a chance additional elements of rare disease policy will be addressed more comprehensively when the Ministry develops Peru’s national plan, which is one the other mandates in the final regulation.

Argentina has two resolutions and one decree on the books to implement national rare disease policy. One resolution combines rare diseases with congenital abnormalities and establishes a national program within the Health Secretary [17]. The decree on the other hand is focused only on implementing the law for rare diseases, but leaves several sections blank in terms of describing actions to be taken by the government [18]. This regulatory ambiguity may have slowed concerted efforts to address rare diseases comprehensively in Argentina, although a second resolution recently issued constitutes a national Advisory Board that could lead to more alignment [19, 27]. In Chile, the Ricarte Soto law folds rare diseases into the broader “high-cost diseases” with no specific regulation of rare diseases other than providing diagnosis and treatment coverage for some. The law does provide some new protections for clinical research subjects [28], but again this is not specific to the rare disease patient community.

In our analysis, the opportunity for regulation is perhaps greatest in Mexico where rare diseases is established in national law and some initiatives exist, but where government coordination and oversight has been minimal. There is a strong case to be made in Mexico for a unified national plan that pulls together the work of the new rare diseases commission of the national health council with access programs established for some patients covered by the Institute for Social Security and Services for State Workers (ISSSTE) and Seguro Popular, for example. Rare disease patient advocacy organizations in Mexico have previously suggested a national plan [29], which has also been requested of the Secretary of Health by the previous Mexican national Senate [30].

### Health system incorporation

A national plan in Mexico might help address the difficult incorporation process for orphan drugs for rare diseases, which at the moment leads to uncertain and inconsistent patient access to needed treatments. Most orphan drugs do not make it through the laborious process for national reimbursement in Mexico despite prior approval by the drugs regulatory agency (the Federal Commission for the Protection Against Sanitary Risk, or COFEPRIS). Even when successful, few of these proceed to make it onto individual payer formularies, such as for the Mexican Social Security Institute (IMSS), by far the largest payer in Mexico [31]. Drug purchases without formulary approval can strain the budgets of even the largest healthcare payers making this approach unsustainable over time. A different approach in Mexico could be for the government to consider distinct rules for rare disease medicines in its national formulary process and to make formulary decisions equally binding across all public payers.

Like in Mexico, the incorporation process in Brazil has traditionally been difficult for orphan drugs, though there have been recent improvements as listed in Table 4. A number of rare disease medicines were accepted for SUS inclusion at the end of 2017 with several more in 2018, along with an increase in the number of required PCDTs [32–35]. Key drivers of these recent incorporations in Brazil may have been to secure better prices and limit patient access via lawsuits. Brazil’s National Sanitary Surveillance Agency (ANVISA) has also recently rolled out new processes designed to speed review and approval of orphan drugs [36]. If both trends are maintained--speedier approval along with greater possibility for SUS inclusion--access for patients with rare diseases should improve over time.

**Table 4:** Brazil SUS Incorporations and PCDTs – National Committee for Technology Incorporation (CONITEC) Recommendations 2016–2019

Year	Treatment	Rare Disease	Decision	Date
2016 [32]	–	–	–	–
–	Tobramycin	Cystic Fibrosis	Incorporate	10/27/2016
–	Golimumab	Psoriatic Arthritis	Incorporate	4/12/2016
–	Pancrelipase	Cystic Fibrosis	Exclude	1/18/2016
2017 [33]	–	–	–	–
–	Idursulfase	MPS II	Incorporate	12/20/2017
–	Somatropin	Turner Syndrome and Hypopituitarism	Incorporate	11/3/2017
–	Laronidase	MPS I	Incorporate	9/4/2017
–	PCDT	Cystic Fibrosis (Pancreatic Insufficiency)	Approve	9/4/2017
–	PCDT	Cystic Fibrosis (Pulmonary)	Approve	9/4/2017
–	Alfataliglicerase	Gaucher Disease	Increase use	7/12/2017
–	PCDT	Psoriatic Arthritis	Approve	7/19/2017
–	PCDT	Gaucher Disease	Approve	6/27/2017
2018 [34]	–	–	–	–
–	Pirfenidone	Idiopathic Pulmonary Fibrosis	Not Incorporate	12/26/2018
–	Nintedanib	Idiopathic Pulmonary Fibrosis	Not Incorporate	12/26/2018
–	Zoledronic Acid	Paget’s Disease	Incorporate	12/21/2018
–	Galsulfase	MPS VI	Incorporate	12/20/2018
–	Elosulfase Alfa	MPS IVa	Incorporate	12/20/2018
–	Sapropterin	Phenylketonuria	Incorporate	12/17/2018
–	Eculizumab	Paroxistic Nocturnal Hemoglobinuria	Incorporate	12/17/2018
–	Agalsidase (Alfa and Beta)	Fabry’s Disease	Not Incorporate	12/17/2018
–	Eltrombopag Olamine	Idiopathic Thrombocytopenic Purpura	Incorporate	12/12/2018
–	Romiplostim	Idiopathic Thrombocytopenic Purpura	Not Incorporate	12/12/2018
–	PCDT	Autoimmne Hemolytic Anemia	Approve	12/10/2018
–	PCDT	Psoriatic Arthritis	Approve	11/5/2018
–	PCDT	Familial Amyloid Polyneuropathy	Approve	10/10/2018
–	PCDT	MPS II	Approve	5/25/2018
–	PCDT	Turner Syndrome	Approve	5/24/2018
–	PCDT	Autoimmune Hepatitis	Approve	5/24/2018
–	PCDT	Biotinidase Deficiency	Approve	5/24/2018
–	PCDT	MPS I	Approve	4/18/2018
–	PCDT	Wilson Disease	Approve	4/9/2018
–	PCDT	Sickle Cell Disease	Approve	2/22/2018
–	Certolizumab	Psoriatic Arthritis	Not Incorporate	1/25/2018
–	Ustekinumab	Psoriatic Arthritis	Not Incorporate	1/25/2018
–	Tafamidis	Familial Amyloid Polyneuropathy	Incorporate	1/18/2018
2019 (Up to 4/1/19) [35]	–	–	–	–
–	Eftrenonacog Alfa	Hemophilia B	Not Incorporate	2/22/2019
–	Efmoroctocog Alfa	Hemophilia A	Not Incorporate	2/22/2019
–	Secukinumab	Psoriatic Arthritis	Incorporate	1/21/2019
–	PCDT	Acromegaly	Approve	1/14/2019

Of course, health care budgets will need to keep pace with these trends, which is one of the long-term questions for Chile’s Ricarte Soto law. Current coverage under the law is outlined in Table 5. The program covers certain treatments and services for 18 high-cost diseases, 8 of which are rare diseases, and in July 2019 the Ricarte Soto law will begin to cover treatments for an additional 7 rare diseases and conditions [37]. Still, with hundreds of solicitations for program coverage each year, it will be difficult for the Chilean government to meet the public’s expectations over time [38]. The budget for Ricarte Soto has doubled since implementation, but up to December 2018 the program had served a total of only about 13,000 patients with approximately 8000 still receiving treatment [39].

**Table 5:** Ricarte Soto Law – Covered Rare Diseases [37]

	Disease/Condition	Treatment
Current Coverage		
1	Mucopolysaccharidosis I	Laronidase
2	Mucopolysaccharidosis II	Idursulfase
3	Mucopolysaccharidosis VI	Galsulfase
4	Tyrosinemia	Nitisinone
5	Gaucher Disease	Taliglucerase or Imiglucerase
6	Fabry Disease	Agalsidase Alfa or Beta
7	Pulmonary Arterial Hypertension (Group 1)	Ambrisentan, Bosentan, or Iloprost
8	Hereditary Angioedema	C1 Esterase Inhibitor
Additions for Coverage beginning July 1, 2019		
9	Epidermolysis Bullosa (dystrophic or junctional)	Treatment Pack
10	Amyotrophic Lateral Sclerosis (moderate to severe)	Technical Assistance and Devices
11	Gastrointenstinal Stromal Tumor (GIST)	Imatinib or Sunitinib
12	Psoriatic Arthritis (moderate to severe, refractory)	Golimumab, Etanercept, Adalimumab, or Secukinumab
13	Ulcerative Colitis (moderate to severe, refractory)	Golimumab or Adalimumab for moderate; Infliximab for severe
14	Myelofibrosis (primary and secondary to other myeloproliferative neoplasms)	Ruxolitinib
15	Huntington’s Disease (chorea)	Tetrabenazina

Health systems in Colombia, Argentina, and Peru so far lack specific processes for prioritizing access to rare disease treatments. In Peru, the final regulation mandates the health ministry build a list of rare diseases and their respective high-cost treatments, but stops short of describing reimbursement parameters. In Colombia and Argentina, litigation for access has been described as routine [40, 41]. All three governments are focused on price control, centralized purchasing, and new medicine evaluation agencies to limit budget shock and to prioritize value. Still, most of these initiatives are new and in any case are not specific to medicines for rare diseases.

### Care delivery

Many would consider Peru’s final regulation to be a missed opportunity for the government to organize and sanction a network of reference centers for the treatment and management of rare disease patients. After all, previous drafts of the regulation included this idea [42], plus there are a few institutions in Peru that lead efforts in rare disease research, diagnosis, and treatment [43]. The omission is all the more glaring given that the final regulation in Peru mandates more research in rare diseases, as well as more training opportunities for health personnel. Diagnostic and treatment reference centers could be something to advocate for in Peru as the health ministry begins to develop a national plan for rare diseases.

In Argentina the greatest current opportunity is for the government and patient advocates to work together on a mapping of the country’s infrastructure with regard to the care and management of rare disease patients. This is one of the few areas in which there is general alignment between Argentina’s national law and its implementing regulations. Also, patient advocacy organizations like the Federation of Rare Diseases in Argentina (FADEPOF) have experience in conducting surveys of the rare disease community [44], while the government has implemented some rare disease training for physicians in public hospitals across the country [45]. Data collected from these kinds of efforts could form the basis for the rare disease infrastructure mapping required by law.

Colombia’s Health Ministry issued a resolution in 2018 to establish diagnostic and treatment reference centers among existing health care institutions, as well as to sketch how these reference centers should be networked. A detailed manual was made available for any institution seeking reference center status [25]. To date, there has been little news as to whether or how many reference centers are being designated. Still, this effort to better organize the delivery of care to rare disease patients is promising, especially given Colombia’s focus on mandated systems for tracking rare diseases and on the specified rights of these patients enshrined in Circular 011 from the Colombian Health Superintendency [46].

In Mexico, ISSSTE and Seguro Popular have adopted programs to allow some patients with rare diseases to access treatments and services [47, 48]. However, coverage is highly selective and continuously subject to administrative barriers that result in significant access delays. Moreover, if the new Administration has its way, Seguro Popular for the most vulnerable populations could be absorbed by IMSS, creating new uncertainty for rare disease patients without any type of health insurance coverage [49]. Given Mexico’s concentration of medical specialty expertise and services in major urban areas of the north, west, and center of the country, there is opportunity in building strong regional networks of diagnosis and treatment reference centers for rare diseases. In fact, mapping the country’s existing medical infrastructure for rare diseases should be a future policy goal in Mexico.

Ordinance 199 in Brazil has a large focus on care delivery via rare disease reference centers. Throughout 2016, seven centers received full authorization from the Ministry of Health, while another ten hospitals had been accredited to provide services for rare disease patients but were awaiting full authorization. Nevertheless, the relative scarcity of rare disease PCDTs means the services provided by these reference centers are limited. In fact, most fully authorized centers in Brazil focus on birth defects, inborn errors of metabolism, and/or intellectual disabilities. In addition, most of the reference centers able to provide rare disease services of any kind are still concentrated in the major urban centers of Sao Paulo and Rio de Janeiro [12]. It remains to be seen if recent rare disease drug incorporations into SUS leads to a greater range of rare diseases being attended to in Brazilian reference centers.

In Chile, the process established by the Ricarte Soto law requires an officially sanctioned clinical treatment guideline prior to consideration of a disease and its medicine for coverage [37]. Perhaps the assumption was that the new law would incentivize the production of such treatment guidelines, and in doing so raise quality standards of care across the board. But it appears this has not been a priority for the Health Ministry as it implements the law. The focus has been on diseases and conditions where care standards already exist, instead of on new treatment guidelines, which may put lesser-known rare diseases at a relative disadvantage for coverage within the high-cost disease category [50].

### Patient engagement

Patient engagement comes in many forms and at different levels. Some of the major rare disease patient organizations in the six countries are listed in Table 6. A government’s willingness to engage with patient advocates can be an important measure of a country’s sustainable policy framework in rare diseases. For example, laws can establish a national recognition day for rare diseases to provide non-governmental groups an important platform for public awareness. Similarly, regulations can mandate that patient advocates be included in rare disease policy, governance and oversight entities.

**Table 6:** Rare Disease Patient Advocacy Organizations in Six Latin American Countries

Argentina	Federación Argentina de Enfermedades Poco Frecuentes (FADEPOF)	http://fadepof.org.ar/
Brazil	Instituto Vidas Raras	http://www.vidasraras.org.br/site/
Chile	Fundación de Enfermedades Lisosomales Chile (FELCH)	http://www.fundacionfelch.cl/
Chile	Federación Chilena de Enfermedades Raras (FECHER)	https://twitter.com/fecher_cl
Colombia	Federación Colombiana de Enfermedades Raras (FECOER)	http://www.fecoer.org/
Colombia	Observatorio Interinstitucional de Enfermedades Huerfanas (ENHU)	https://twitter.com/EHuerfanasCo
Mexico	Organización Mexicana de Enfermedades Raras (OMER)	http://omer.org.mx/
Mexico	Federación Mexicana de Enfermedades Raras (FEMEXER)	http://www.femexer.org/
Peru	Coalición Peruana de Enfermedades Poco Frecuentes (COPEPOFRE)	http://www.esperantra.org/index.php/copepofre
Peru	Federación Peruana de Enfermedades Raras (FEPER)	http://feperperu.blogspot.com/

In Brazil, rare disease Ordinance 199 includes no provisions for regular engagement with patient advocates in spite of mandating increased public awareness about rare diseases. Rare disease patient advocates and their organizations are involved in Brazilian policy efforts in terms of pending legislative proposals [51]. Advocates also take part voluntarily in the SUS incorporation process when medicines that serve their respective communities are up for consideration [52]. Still, the opportunity is great for the government of Brazil to more officially recognize the expertise and engage more formally with patient advocates.

There is also opportunity in Peru for the health ministry to capitalize on growing expertise from the patient advocacy community. There is a Peruvian Federation of Rare Diseases (FEPER) made up of 17 different rare disease patient-based associations [53]. In addition, 23 rare disease patient-based associations in 2016 formed the Peruvian Coalition for Rare Diseases (COPEPOFRE) to collaborate on rare disease and orphan drug policy advocacy [54]. In 2012, shortly after Peru passed its national law on rare diseases, an annual day of recognition was established and advocates have been able to use that opportunity each year to raise public awareness [55]. Unfortunately, the final regulation implementing Peru’s national rare diseases law avoids both patient engagement and public awareness as priorities even though earlier drafts had included these as priorities.

In Mexico, patient advocacy organizations were instrumental in establishing the program in rare diseases at ISSSTE [56], but were not included in any of the official processes when Mexico’s General Health Council established its National Rare Diseases Commission in 2017 [57]. Several organizations have been effective in the larger urban centers of the country and some of them work with members of the national Congress to raise awareness and seek policy solutions. Nevertheless, questions have arisen as to whether the current Administration is open to input from civil society, in general, which could have an impact on rare disease patient advocacy in Mexico [58].

It is noteworthy for the region that in Chile patient advocates participate officially in the different processes of the Ricarte Soto program as high-cost diseases and medicines are considered for coverage. The law is explicit in that patient and public input be considered [38]. Nevertheless, participation in official decision making is limited to only a few advocates at any one time. Also, strict rules on conflict-of-interest govern selection, which can unnecessarily preclude direct participation by patient advocates who have the most experience in the field. This selection bias can be especially acute in the field of rare diseases where the number of advocates for each disease is fewer than for more common high-cost health conditions, further compounding the relative disadvantage of rare diseases within the Ricarte Soto program. It has also been reported that the Ricarte Soto law has served to divide the rare disease patient community in Chile [59].

In Colombia, rare disease patient advocates were instrumental in the adoption of Law 1392 and the strong focus on ensuring rare disease patients have certain inalienable rights and protections within the health system [60]. A voluntary working group on rare diseases within the Ministry of Health has been meeting regularly since 2012 and includes patient advocacy representatives [61]. At the municipal level, in June 2014 Bogota’s mayor issued Resolution 1147 establishing a technical coordinating committee that meets regularly to design and implement strategies to address the needs of patients with rare diseases. The resolution included a mandate to include on the committee a representative from the Colombian Federation of Rare Diseases (FECOER), which set an important precedent for patient advocates to work closely with city government officials and physicians on rare disease policy [62].

Argentina’s rare disease law includes a mandate for a multidisciplinary advisory board to help the government develop and promote initiatives. Only in February 2019 did this advisory board become officially constituted with rare disease patient advocates to be represented by FADEPOF, among others [63]. While an important step forward, the Federation has in fact articulated several initiatives over the years for the Argentine government to implement, including both the basis for an official disease registry and a detailed national plan for rare diseases [64]. A rare diseases registry is currently being piloted by the government as part of Argentina’s National Health Information System (SIISA), which includes other data registries [65]. With the work of the advisory board soon underway, it remains to be seen if the authorities will now be more open to the expertise and resources on offer by civil society [66].

## Conclusions

This paper summarizes rare disease public policy and the current context for implementation in Argentina, Brazil, Chile, Colombia, Mexico, and Peru. Five policy dimensions were chosen to facilitate analysis among the six countries: national laws, national regulations, health system incorporation of rare disease treatments, care delivery, and patient engagement. Based on these dimensions, several opportunities were identified for strengthening rare disease policies. Here we summarize these opportunities for each country.

In Argentina, almost eight years have passed since passage of the rare diseases national law and four years since implementing regulations were adopted. Regulatory ambiguity is likely responsible for at least some of the lack of concrete action on rare diseases and greater clarity via Congressional action or by the Administration is an important opportunity. Another important opportunity is a mapping of the country’s infrastructure with regard to the care and management of rare disease patients. Both patient advocates and the government have relevant expertise in this area.

In Brazil, there is opportunity with the many legislative proposals in Congress to pass a national law that serves to fill gaps and modernize the rare diseases Ordinance 199 from 2014. Much progress has recently been made by both ANVISA and CONITEC in terms of new regulatory pathways for orphan drug review, PCDTs, and SUS incorporation recommendations. There is opportunity in ensuring these decisions translate quickly to the benefit of rare disease patients in SUS specialized care units and authorized reference centers where multidisciplinary teams of health professionals manage care.

In Chile, it is increasingly apparent that while the Ricarte Soto Law has benefitted some rare disease patients with coverage and access to treatment, the program is insufficient in terms of unmet medical need and perhaps unsustainable over time. There is significant opportunity in Chile to pursue laws and regulations specific to rare diseases, which at the very least would facilitate productive interactions among a highly fragmented rare disease patient advocacy community.

Colombia’s rare disease law is the oldest of the six countries—adopted in 2010—and has an explicit emphasis on protecting the rights of patients with rare diseases. Rare disease advocates have been able to exercise those rights whenever additional health care reforms are proposed. Nevertheless, Colombia’s implementing regulations have focused mostly on disease and patient identification, with the recent regulation to establish authorized rare disease reference centers a step forward. The opportunity in Colombia is for the government to meaningfully address the mostly negative experience of rare disease patients in accessing quality care.

Mexico benefits from a concentration of medical specialty expertise and services in several large urban areas of the country. There is opportunity for the government to build capable regional networks of diagnosis and treatment reference centers for rare diseases. As an initial step, a map of Mexico’s existing medical infrastructure for rare diseases should be a future policy goal. There is also significant opportunity for a different approach with distinct rules for rare disease medicines in its national formulary process. The rare diseases commission of Mexico’s National Health Council should be encouraged to work with patient advocates to link their initial efforts at building a rare disease registry to other policy processes governing access to rare disease medicines and services.

The rare diseases law and final regulation in Peru mandates the development of an action plan, which can be an important means of building consensus and benchmarking progress on the country’s policy objectives. When updated regularly, national plans can keep stakeholders focused on respective priorities and governments can be held more accountable for results. The opportunity in Peru is for the government and advocates to work together to ensure key elements omitted from the final regulation—such as diagnostic and treatment reference centers or patient engagement—are reinserted as priorities as the health ministry begins to develop a national plan for rare diseases.

Finally, we note that recent landmark Presidential elections in Brazil, Mexico, and Colombia, as well as one upcoming in Argentina are likely to have an impact on health care plans and priorities, and by extension, government programming in rare diseases. It is imperative that rare disease leaders in each country continue their work to educate newly elected or appointed stakeholders on the progress achieved as well as pending opportunities to build and advance policy frameworks on behalf of rare disease patients. Readers are encouraged to review the Asia-Pacific Economic Cooperation’s (APEC) Rare Disease Network Action Plan launched in November 2018 to provide a framework for continued rare disease policy action by each of its 21-member economies [67]. In addition, continued in-depth research and analysis will help our respective stakeholder communities across Latin America identify salient opportunities, build consensus, and make significant improvements moving forward.

### Study limitations

We chose five dimensions to analyze rare disease policy in six Latin American countries based on patient advocate views supplemented by online research. Additional national or local laws such as rules on newborn screening, human rights, and disabilities were not considered in this analysis but can have significant impact on the welfare of the community of rare disease patients. Another study limitation is heavy reliance on patient advocate perspectives, which can bias the findings in this paper especially as these views were collected during the course of several face-to-face patient advocate meetings and telephone discussions in 2016, 2017, and 2018. Further structured research to validate patient advocate views and that include the experience and perspectives of additional stakeholder groups would help elucidate rare disease policy implementation across Latin America. Finally, we did not employ any technical platform to conduct the desktop research, including for the literature search. This introduces certain subjective bias into the findings as we may have missed or overlooked publications or other key resources relevant to the topics discussed in this paper.

## Data Availability

The datasets used and/or analyzed during the current study available from. the corresponding author on reasonable request.

## Abbreviations

ANVISA: Brazil’s National Sanitary Surveillance Agency
APEC: Asia-Pacific Economic Cooperation
COFEPRIS: Mexico’s Federal Commission for the Protection Against Sanitary Risk
CONITEC: Brazil’s National Committee for Technology Incorporation
COPEPOFRE: Peruvian Coalition for Rare Diseases
FADEPOF: Federation of Rare Diseases in Argentina
FECOER: Colombian Federation of Rare Diseases
FEPER: Peruvian Federation of Rare Diseases
IMSS: Mexican Social Security Institute
ISSSTE: Mexico’s Institute for Social Security and Services for State Workers
PCDT: Brazil’s Clinical and Therapeutic Protocols
RENETSA: Peru’s National Network for Health Technology Evaluation
SISPRO: Colombia’s Integrated Information System for Social Protection
SUS: Brazil’s Unified Health System
